# Lymphangioma circumscriptum of the left thigh in a pediatric patient: a radiological diagnosis correlated with ultrasonography

**DOI:** 10.11604/pamj.2026.53.112.49944

**Published:** 2026-03-04

**Authors:** Riya Yadav, Pratap Singh Parihar

**Affiliations:** 1 Department of Radio-Diagnosis, Datta Meghe Institute of Higher Education and Research, Wardha, Maharashtra, India

**Keywords:** Pediatric lymphatic malformation, thigh cystic lesion, ultrasonography correlation

## Image in medicine

A 12-year-old female presented to the dermatology department with a history of gradually progressive swelling over the posterolateral aspect of the left thigh for the past six months. The overlying skin appeared normal without discoloration or ulceration. Neurovascular examination of the limb was normal. Contrast-enhanced magnetic resonance imaging (MRI) of the left thigh revealed multiple, well-defined cystic lesions within the subcutaneous plane of the posterolateral aspect of the left thigh. These lesions appeared hyperintense on T2 and T2 fat-suppressed sequences, hypointense on T1, and showed minimal thin peripheral enhancement following contrast administration. No diffusion restriction on DWI images. The underlying muscles, myofascial planes, femoral cortex, and neurovascular bundles were intact. The lesions collectively measured approximately 2.2 x 2.6 x 3 cm (AP X TR X CC). Ultrasonography was done for correlation of the left thigh, which shows a well-defined, multiloculated cystic lesion confined to the subcutaneous plane. The lesion showed multiple thin internal septations with anechoic contents and no significant internal vascularity on Doppler. Based on the combined radiological findings, the final diagnosis of lymphangioma circumscriptum of the left thigh was made. The patient was referred to pediatric surgery for further evaluation. Surgical excision was planned due to progressive enlargement and cosmetic concerns. Complete excision of the superficial lesion was performed. Histopathological examination confirmed the diagnosis of lymphangioma circumscriptum of the left thigh. The postoperative period was uneventful. At short-term follow-up (3months), there was satisfactory wound healing with no evidence of recurrence.

**Figure 1:**
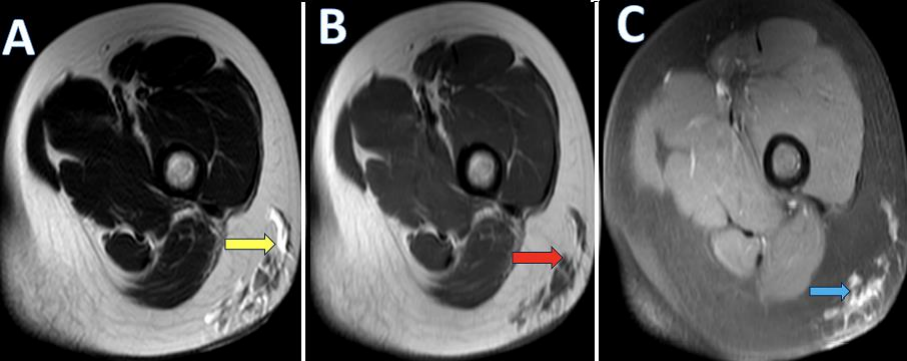
axial magnetic resonance imaging images of the left thigh demonstrating multiple well-defined cystic lesions in the subcutaneous plane of the posterolateral aspect of the thigh (arrows); A) T2-weighted image showing hyperintense cystic lesions (yellow arrow) in the subcutaneous tissue; B) T1-weighted image demonstrating hypointense signal intensity of the same lesions (red arrow); C) post-contrast T1 fat-suppressed image showing minimal thin peripheral enhancement of the cystic lesions (blue arrow)

